# Editorial: Nanofibrous biomaterials for biomedical applications

**DOI:** 10.3389/fbioe.2023.1279471

**Published:** 2023-08-30

**Authors:** Botao Song, Zhenhua Chen, Haiyong Ao, Meili Zhang

**Affiliations:** ^1^ Northwest University, Xi’an, Shaanxi, China; ^2^ Jinzhou Medical University, Jinzhou, Liaoning, China; ^3^ East China Jiaotong University, Nanchang, Jiangxi, China; ^4^ The University of Queensland, Brisbane, QLD, Australia

**Keywords:** nanofiber, electrospinning, bioactive, biomaterials, tissue regeneration

Nanofibers represent a distinctive class of biomaterials characterized by their high similarity to the 1D structure of the extracellular matrix, coupled with their high porosity and large specific surface area. These unique features bestow the nanofiber biomaterials with great potential in tissue regeneration and disease diagnosis. Owing to great demand on such promising biomaterials, we are delighted to release the Research Topic of “*Nanofibrous Biomaterials for Biomedical Applications*.” This Research Topic has aroused the interest of numerous relevant experts and gathered many cutting-edge research advances on nanofiber biomaterials.

Yamaura et al. fabricated novel peptide amphiphile (PA) nanofibers loaded with transforming growth factor beta-1 (TGF-β1) inhibitor losartan by a self-assembly strategy for cartilage regeneration. The results revealed that losartan exhibited a sustained release from the PA nanofibers over 2 weeks. Furthermore, the losartan-loaded PA nanofibers showed no cytotoxicity and demonstrated the capability to promote ATDC5 cell proliferation. The *in vitro* studies additionally indicated that PA nanofibers loaded with losartan had significant anti-inflammatory, anti-degenerative, and cartilage regenerative capacity.

Zheng et al. investigated the interaction between electrospun yarn scaffolds and three types of cells [bone marrow mesenchymal stem cells (BMSCs), adipose-derived stem cells (ADSCs), and articular chondrocytes] to evaluate their potential for meniscus tissue engineering in the absence of growth factors. The electrospun yarn scaffolds showed a pattern of aligned fiber structure, closely resembling the natural structure in the native meniscus. CCK-8 assay results demonstrated that BMSCs, ADSCs, and chondrocytes exhibited robust proliferation over 14 days, and live/dead staining further proved the good cytocompatibility of the scaffolds. Three-dimensional confocal images revealed that the three types of cells could grow along the thickness direction of the electrospun fiber yarn. Moreover, compared with BMSCs and ADSCs, a significant shape change from spindle shape to spherical shape was observed in the articular chondrocytes. The results of Alcian blue staining revealed a substantial presence of glycosaminoglycan (GAG)-rich extracellular matrix in the chondrocyte group signifying the formation of a cartilage lacuna structure.

Zheng et al. reported a hydrogel loaded with a major active constituent of epimedium, icariin, and light-responsive carbon nanofibers for treating periodontitis. Under near-infrared (NIR) irradiation, the composite hydrogel showed high efficacy in eradicating both *S. aureus* and *E. coli. In vitro* results also demonstrated that the composite hydrogel promoted M1 to M2 shift in RAW264.7 cells by suppressing the expression of inflammatory factor IL-6 and reactive oxygen species (ROS) and concurrently up-regulating the expression of anti-inflammatory factor IL-10. The composite hydrogel additionally significantly promoted the osteogenic differentiation of BMSCs when exposed to NIR. The *in vivo* results showed that the composite hydrogel alleviated the destruction of alveolar bone. Taken together, the combination of therapeutic drug and photothermal carbon nanofibers within the hydrogel offers a new approach for the management of periodontitis.

Ding et al. developed a hydrogel using a blend of polyvinyl alcohol (PVA) and sodium alginate (ALG), which was loaded with silver (Ag) in the nanofiber form for the potential application in wound healing. The Ag based hydrogel showed excellent photothermal effect with the temperature reaching 45.4°C after 10 min light irradiation. In addition, the hydrogel demonstrated favorable compression behavior. More importantly, ascribed to the encapsulation of Ag, the composite hydrogel showed significant antibacterial effect on *MRSA* and *E. coli*.

Guo et al. proposed photothermal hyaluronic acid (HA) modified multiwalled carbon nanotubes (MWCNT-HA) for accelerating the apoptosis of nasopharyngeal carcinoma cells. The MWCNT-HA showed an obvious photothermal behavior with the temperature reaching 56°C after laser treatment. The *in vitro* studies confirmed that MWCNT-HA inhibited the proliferation of CNE-1 cells under NIR irradiation. MWCNT-HA could induce cell death by suppressing the mitochondrial pathway in CNE-1 cells.

Ren et al. proposed a novel method by using ionic liquids to fabricate hydroxyapatite (HA) nanofibers, and the HA nanofibers were then used as a filler to improve the mechanical properties of a hydrogel. The ultralong HA nanofibers were fabricated and characterized by scanning electron microscope (SEM) and X-ray diffraction (XRD). The underlying formation mechanism of the HA nanofibers was then clarified. Both the stretching and compressing experiment results indicated that the HA nanofibers modified hydrogel showed better mechanical performance than that of the pure hydrogel.

Tang et al. reported honokiol-loaded titanium dioxide nanotubes (HNK-TNTs) for the treatment of tongue cancer. The hollow structured titanium dioxide nanotubes were prepared by an anodic oxidation method, followed by adsorption of the drug honokiol. The *in vitro* results showed that HNK-TNTs inhibited the proliferation and migration of CAL-27 cells, and promoted CAL-27 cells apoptosis by increasing the expression levels of apoptosis factors of Bax and Fas.

Based on these publications in the Research Topic, we can continue to observe that nanofiber biomaterials have potential for versatile applications, encompassing cartilage regeneration, tissue engineering, bone repair, wound healing, antitumor treatment, antimicrobial interventions, and mechanical strength enhancement. This compilation of articles will provide readers with a broad guidance and ignite their interest in the field of nanofiber biomaterials.

